# Blockade of the BLT1-LTB_4_ axis does not affect mast cell migration towards advanced atherosclerotic lesions in LDLr^−/−^ mice

**DOI:** 10.1038/s41598-022-23162-4

**Published:** 2022-11-01

**Authors:** Marie A. C. Depuydt, Femke D. Vlaswinkel, Esmeralda Hemme, Lucie Delfos, Mireia N. A. Bernabé Kleijn, Peter J. van Santbrink, Amanda C. Foks, Bram Slütter, Johan Kuiper, Ilze Bot

**Affiliations:** Division of BioTherapeutics, Leiden Academic Centre for Drug Research, Leiden University, Leiden, The Netherlands

**Keywords:** Mast cells, Atherosclerosis, Inflammation

## Abstract

Mast cells have been associated with the progression and destabilization of advanced atherosclerotic plaques. Reducing intraplaque mast cell accumulation upon atherosclerosis progression could be a potent therapeutic strategy to limit plaque destabilization. Leukotriene B_4_ (LTB_4_) has been reported to induce mast cell chemotaxis in vitro. Here, we examined whether antagonism of the LTB_4_-receptor BLT1 could inhibit mast cell accumulation in advanced atherosclerosis. Expression of genes involved in LTB_4_ biosynthesis was determined by single-cell RNA sequencing of human atherosclerotic plaques. Subsequently, Western-type diet fed LDLr^−/−^ mice with pre-existing atherosclerosis were treated with the BLT1-antagonist CP105,696 or vehicle control three times per week by oral gavage. In the spleen, a significant reduction in CD11b^+^ myeloid cells was observed, including Ly6C^lo^ and Ly6C^hi^ monocytes as well as dendritic cells. However, atherosclerotic plaque size, collagen and macrophage content in the aortic root remained unaltered upon treatment. Finally, BLT1 antagonism did not affect mast cell numbers in the aortic root. Here, we show that human intraplaque leukocytes may be a source of locally produced LTB_4_. However, BLT1-antagonism during atherosclerosis progression does not affect either local mast cell accumulation or plaque size, suggesting that other mechanisms participate in mast cell accumulation during atherosclerosis progression.

## Introduction

The mast cell, a cell type of our innate immune system that acts in the first line of defence against pathogens, has been shown to promote the development and progression of atherosclerosis. Upon activation, mast cells secrete the proteases chymase and tryptase and pro-inflammatory cytokines such as IFN-γ, which have been shown to promote atherogenesis and to lead to destabilization of the plaque^1,2^. Systemic activation of mast cells in dinitrophenyl hapten (DNP)-challenged apolipoprotein E (apoE)^−/−^ mice for example resulted in an increased plaque size and incidence of intraplaque haemorrhage^1^, whereas mast cell deficiency was shown to reduce atherosclerotic plaque development^2,3^. In humans, mast cells have been shown to accumulate in advanced and ruptured coronary plaques^4^. More recently, the association of mast cells with disease progression in cardiovascular disease patients was established, as significantly increased serum tryptase levels were observed in patients with acute coronary syndromes^5^ and intraplaque mast cell numbers were seen to increase upon atherosclerotic plaque destabilization^6^. In addition, in that study an independent association of intraplaque mast cell numbers in carotid plaques with the incidence of clinical cardiovascular events was revealed^6^. In patients with systemic mastocytosis, a disease characterized by the accumulation of mast cells in different organs, the prevalence of cardiovascular events was increased, despite a reduction in circulating low density lipoprotein levels^7^. Together, the contribution of mast cells and their activation to atherosclerosis has been well established as has also been reviewed^8,9^, however it remains elusive what factors contribute to mast cell migration towards these plaques.

Apart from various chemokines and cytokines that have been implicated in mast cell migration, lipid mediators have been described to provoke a chemotactic response in mast cells^10,11^. Leukotriene B_4_ (LTB_4_) is a pro-inflammatory lipid mediator well known for its chemotactic effect on myeloid and lymphoid cells^12^. Intracellular biosynthesis of LTB_4_ occurs in a two-step enzymatic reaction in which arachidonic acid is metabolized by 5-lipoxygenase (5-LOX), 5-lipoxygenase activating protein (FLAP) and LTA_4_ hydrolase (LTA_4_H)^13^. Monocytes, macrophages and mast cells are able to release LTB_4_ in response to stimulation with factors such as Complement component 5a (C5a), Interleukin-1 (IL-1), Leukemia Inhibitory Factor (LIF) and Tumor Necrosis Factor α (TNFα)^14^. Synthesis and subsequent release of LTB_4_ by these leukocytes will elicit a directed migration of vascular smooth muscle cells, neutrophils, eosinophils, basophils, monocytes, macrophages, dendritic cells and T cells but also of mast cell progenitors through binding with its receptor BLT1^14–20^. Previous in vitro studies showed an autocrine manner of mast cell migration towards LTB_4_, which mainly recruits mast cell progenitors from the bone marrow as BLT1 expression is significantly reduced upon mast cell maturation^19^.

LTB_4_ and its associated mediators have been suggested to participate in atherogenesis. Heterozygous deficiency of 5-LOX for example resulted in a 95%-decrease in lesion size in Low Density Lipoprotein receptor (LDLr)^−/−^ mice^21^. Moreover, BLT1 deficiency in apoE^−/−^ mice resulted in decreased plaque development^15,22^. Aiello et al*.* showed that antagonism of BLT1 through CP105,696 caused a decrease in the infiltration of monocytes into the lesions as well as reduced monocyte activation^23^. In these studies however, the authors primarily assessed initial lesion development and the number of mast cells in these lesions was not assessed.

As mast cell numbers particularly accumulate in advanced atheromatous human plaques, it may be of therapeutic interest to identify whether the LTB_4_-BLT1 axis is involved in mast cell recruitment to advanced atherosclerosis. In our study, we thus first aimed to determine whether cells in the advanced plaque are able to produce LTB_4_ and next investigated whether LTB_4_ participates in the chemotaxis of mast cells towards pre-established atherosclerotic plaques and would thereby contribute to further progression of the disease. Here, we administered the BLT1-antagonist CP105,696 to LDLr^−/−^ mice with pre-existing atherosclerosis and analysed its effect on plaque progression. In this study, treatment with CP105,696 resulted in reduced splenic myeloid cell content, but did not affect plaque morphology and mast cell accumulation in advanced atherosclerosis.

## Results

### Single cell RNA sequencing reveals expression of ALOX5 on human plaque mast cells

**Figure 1:**
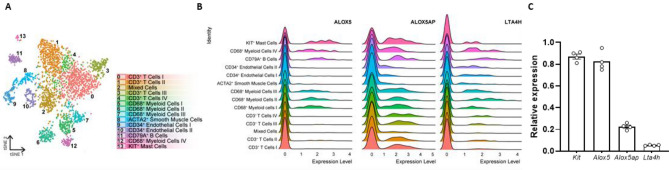
Expression of *ALOX5*, *ALOX5AP* and *LTA4H* in both human atherosclerotic plaque cells and murine bone-marrow derived mast cells. (**A**) Single-cell RNA sequencing of human atherosclerotic plaque cells revealed 14 distinct cell clusters, including one mast cell cluster (cluster 13)^28^. In these clusters, we measured the expression of (**B**) *ALOX5, ALOX5AP* and *LTA4H*. (**C**) Expression of *Kit*, *Alox5*, *Alox5ap* and *Lta4h* in murine bone-marrow derived mast cells. Human data n = 18; murine data: n = 4. Data represent mean ± SEM.

Blockade of mast cell recruitment to atherosclerotic lesions may be a promising intervention target to reduce plaque destabilization. Mast cells have previously been described to be involved in their own recruitment^11,24^, in which LTB_4_ can act as an autocrine chemoattractant in multiple diseases^19,20,25,26^. Moreover, transcriptome analysis of ex vivo skin mast cells using deep-CAGE sequencing revealed expression of *ALOX5* (5-LOX), *ALOX5AP* (FLAP) and *LTA4H* (LTA_4_H), the rate-limiting enzymes needed for biosynthesis of LTB_4_^27^. Yet, it remains unknown whether plaque cells, among which mast cells, can produce LTB_4_ in atherosclerotic lesions. In a previous study, we performed single-cell RNA sequencing on human atherosclerotic plaques obtained from carotid endarterectomy surgery from 18 patients^28^. Fourteen different cell clusters were found, of which one distinctly represented mast cells (cluster 13, Fig. 1A). Within this data set, we analysed the expression of the aforementioned genes involved in LTB_4_ biosynthesis. Indeed, we observed expression of *ALOX5*, *ALOX5AP* and *LTA4H* in several myeloid cell populations, including mast cells, from human atherosclerotic plaques. Whereas *ALOX5AP* and *LTA4H* was ubiquitously expressed by all leukocytes, mast cells showed the highest expression of *ALOX5* (Fig. 1B). To assess whether these genes are also expressed in murine atherosclerotic plaques, we examined a publicly available murine single-cell RNA sequencing data set by Cochain et al.^29^. In this study, single-cell RNA sequencing was performed on CD45^+^ cells isolated from aortas from LDLr^−/−^ mice fed a chow diet, 11 weeks high fat diet and 20 weeks high fat diet, representing respectively the healthy aorta, early atherosclerotic and advanced atherosclerotic aortas. Integrating data from all three mouse models revealed 17 different clusters (Figure S1A–B). *Alox5*, *Alox5ap* and *Lta4h* were mainly found in the *Cd14*^+^ *Cd68*^+^ *Itgam*^+^ myeloid cell clusters (Figure S1C). Therefore, we isolated all myeloid cells (clusters 0, 2, 3, 4, 6, 8, 13, 15 and 16) and examined whether expression of these three genes differed per plaque stage. *Alox5* was mainly expressed in myeloid cells of healthy aorta’s compared to atherosclerotic aorta’s (Figure S1D). Expression of *Alox5ap* and *Lta4h* showed no differences between plaque stages. Finally, we confirmed the expression of *Alox5*, *Alox5ap* and *Lta4h* in murine bone-marrow derived mast cells (BMMCs) (Fig. 1C). Together, these data imply that LTB_4_ can be produced locally in the plaque and that intraplaque mast cells may be its main source in the human plaque. We next aimed to determine whether blockade of the LTB_4_ receptor affects mast cell migration towards the advanced atherosclerotic lesion.

### CP105,696 treatment significantly lowers percentage of myeloid cells in the spleen

**Figure 2:**
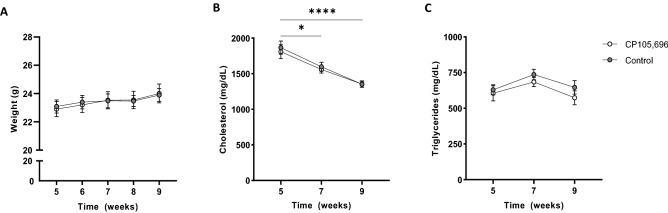
Body weight and plasma lipid levels upon CP105,696 treatment in LDLr^−/−^ mice. (**A**) Body weight of mice was measured weekly during treatment and was not affected by CP105,696 treatment. (**B**) Plasma cholesterol and (**C**) triglyceride concentrations were obtained at t = 5, t = 7 and t = 9. *n* = 15 per group. Data represent mean ± SEM. *p < 0.05; ****p < 0.0001.

Next, we studied the effects of BLT1-antagonist CP105,696 treatment in our LDLr^−/−^ mouse model with pre-existing atherosclerosis (Fig. S2). CP105,696 treatment did not affect total body weight throughout the experiment (t = 9 weeks, Control: 24.0 ± 0.7 g vs. CP105,696: 23.9 ± 0.5 g; Fig. 2A). Serum total cholesterol levels were decreased in both groups during treatment (w5 vs. w9, Control: 1809.7 ± 98.6 mg/dL vs. 1354.8 ± 44.1 mg/dL, *p* = 0.0002 and CP105,696: 1866.2 ± 92.2 mg/dL vs. 1351.9 ± 42.7 mg/dL, *p* = 0.00002), but no differences were found between control and CP105,696 (Fig. 2B). The decline in cholesterol level can be ascribed to the Tween 80 in the solvent as polysorbates were previously reported to induce cholesterol lowering^30^. No differences in serum triglyceride levels were detected (Fig. 2C).

**Figure 3:**
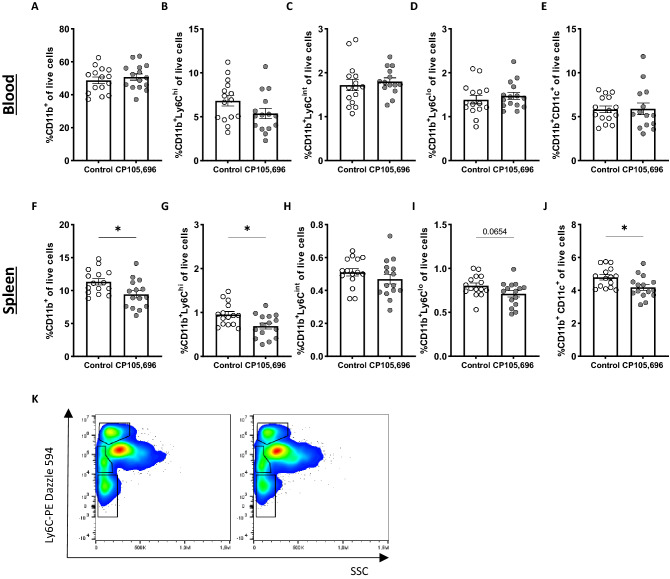
Myeloid cells significantly decreased with BLT1-antagonism in the spleen. Upon sacrifice, blood and spleen were collected and processed to obtain a single cell suspension for flow cytometry analysis. In the blood, the percentage of (**A**) CD11b^+^ myeloid cells, (**B**) Ly6C^hi^ monocytes, (**C**) Ly6C^int^ monocytes, (**D**) Ly6C^lo^ monocytes and (**E**) CD11b^+^CD11c^+^ dendritic cells was not different between the CP105,696 treated and the control group. In the spleen, a significant reduction in the percentage of (**F**) CD11b^+^ myeloid cell content was found with CP105,696 treatment. Specifically, BLT1-antagonism resulted in a decrease in (**G**) the percentage of CD11b^+^Ly6C^hi^ monocytes, no difference in (**H**) the percentage of CD11b^+^Ly6C^int^ monocytes and (**I**) a trend towards a decrease in the percentage of CD11b^+^Ly6C^lo^ monocytes. (**J**) Furthermore, we observed a significant decrease in the percentage of CD11b^+^ CD11c^+^ dendritic cells. (**K**) Representative Flow Cytometry plot of monocytes in the spleen, left: control, right: CP105,696. *n* = 14–15 per group. Data represent mean ± SEM. *p < 0.05.

The BLT1-LTB_4_ axis has previously been described to affect migration of multiple myeloid cells, including monocytes and dendritic cells^31^. To confirm that CP105,696 inhibited the BLT1 receptor in vivo, we used flow cytometry to measure myeloid subsets in blood and spleen. In the circulation, no differences were found in the total percentage of myeloid cells (CD11b^**+**^; Fig. 3A). The percentage of the different monocyte subtypes (CD11b^+^Ly6C^hi^, CD11b^+^Ly6C^mid^ and CD11b^+^Ly6C^lo^) and of CD11b^+^CD11c^+^ cells, which predominantly characterize dendritic cells, also remained unaltered upon treatment (Fig. 3B–E). In the spleen we observed a clear effect of treatment with the BLT1-antagonist. The percentage of total myeloid cells was significantly reduced upon treatment with CP105,696 (Control: 11.3 ± 0.48% vs CP105,696: 9.4 ± 0.56%; *p* = 0.016; Fig. 3F). Both the percentage of inflammatory monocytes (Ly6C^hi^; Control: 0.96 ± 0.067% vs. CP105,696: 0.69 ± 0.069%; *p* = 0.011; Fig. 3G,K) and that of the patrolling monocytes (Ly6C^lo^; Control: 0.80 ± 0.032 vs. CP105,696: 0.71 ± 0.36; *p* = 0.07; Fig. 3I,K) showed respectively a significant decrease and a trend towards a decrease after BLT1 inhibition, whereas Ly6C^int^ monocyte levels did not differ between groups (Fig. 3H,K). Furthermore, we observed a reduced accumulation of CD11b^+^CD11c^+^ dendritic cells in the spleen of treated mice (Control: 4.8 ± 0.16 vs. CP105,696: 4.2 ± 0.17; *p* = 0.016; Fig. 3J).

### Treatment with CP105,696 did not affect plaque progression in advanced atherosclerosis

**Figure 4:**
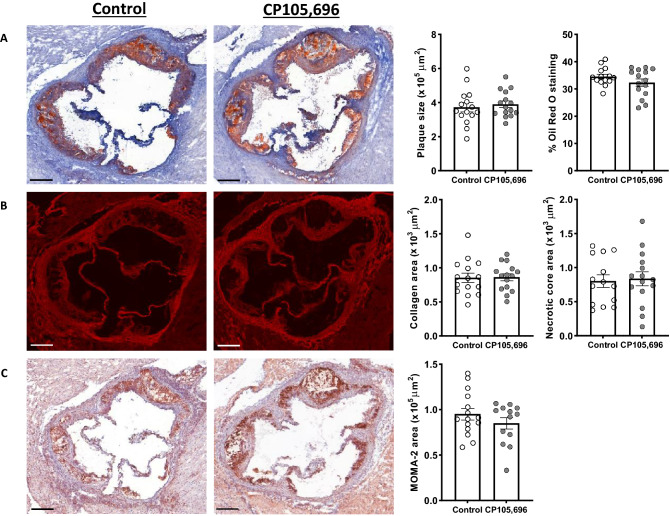
Inhibition of the BLT1-LTB_4_ axis did not alter plaque morphology in advanced atherosclerosis. (**A**) Atherosclerotic plaque size and percentage Oil Red O staining in the plaque did not differ upon treatment with CP105,696 as assessed by Oil-Red O staining of the aortic root. (**B**) Sirius red staining of the aortic root did not reveal differences in plaque collagen and necrotic core area in CP105,696 treated mice versus controls. (**C**) BLT1-antagonism did not affect the MOMA-2 macrophage area in the aortic root. All representative pictures are taken at optical magnification 5x; the bar indicates 200 μm. n = 13–15 per group. Data represent mean ± SEM.

Next, we assessed whether BLT1-antagonism affected the size and morphology of advanced lesions. CP105,696 treatment did not affect aortic root plaque area (Control: 3.7 ± 0.3*10^5^ μm^2^ vs. CP105,696: 3.9 ± 0.2*10^5^ μm^2^; *p* = 0.60) and the percentage of Oil Red O staining in the plaque (Control: 34.5 ± 3.3% vs. CP105,696: 32.3 ± 5.1%; *p* = 0.18; Fig. 4A). The degree of stenosis (Control: 36 ± 2% vs. CP105,696: 39 ± 1%; *p* = 0.233) as well as the total lesion area and plaque volume (Control: 1212 ± 86*10^5^ µm^3^ vs. CP105,696: 1264 ± 64*10^5^ µm^3^; *p* = 0.633) were unaltered by BLT1 antagonism (Figure S3A–C). We also examined collagen content and necrotic core size by Sirius Red staining. Both parameters did not change upon treatment with CP105,696 (Fig. 4B). Furthermore, no differences in macrophage content were observed between the groups (Fig. 4C). In addition, flow cytometry analysis of the atherosclerotic aortic arch did not reveal any differences in the percentage of live CD45^+^ leukocytes of the single cell population and the percentage of total lymphocytes in the CD45^+^ cell population (data not shown) between the groups. Also, the percentage of CD11b^+^ myeloid cells and that of CD11b^+^CD11c^+^ dendritic cells in the CD45^+^ cell population (Figure S4A–C) were not affected by CP105,696 treatment. Thus, BLT1-antagonism did not affect plaque morphology or myeloid cell content in a model of pre-existing atherosclerosis.

### No difference in mast cell accumulation in the aortic root upon BLT1-antagonism

**Figure 5:**
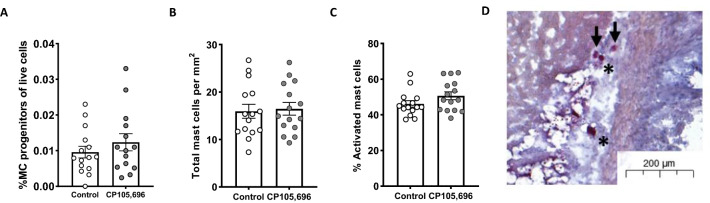
Mast cell accumulation in advanced atherosclerotic plaques (**A**) BLT1-antagonism did not affect the percentage of CD34^+^Lin^-^CD127^-^CD16/32^+^CD117^+^FCεRI^+^ mast cell progenitors in blood. (**B**) The total number of mast cells per mm^2^ perivascular tissue of the aortic root tissue and (**C**) the percentage of activated mast cells did not differ between the CP105,696 treated and control group. (**D**) Representative image of mast cell staining in the aortic root section. Asterix indicates resting mast cells, arrows indicate activated mast cells. n = 14–15 per group. Data represent mean ± SEM.

Subsequently, we aimed to investigate whether LTB_4_ is a chemoattractant for the recruitment of mast cells towards the atherosclerotic plaque. We first assessed the percentage of mast cell progenitors (MCps) in blood and thereby examined whether BLT1-antagonism affected their migration. No differences were observed in CD34^+^Lin^-^CD127^-^CD16/32^+^CD117^+^FCεRI^+^ MCps in blood between groups (Control: 0.010 ± 0.002% vs. CP105,696: 0.012 ± 0.002%; *p* = 0.34; Fig. 5A). We also determined mast cell numbers in the aortic root of treated and non-treated mice. In line with the data on mast cell progenitors, no differences were found in total mast cell numbers (Control: 16.0 ± 1.5 mast cells/mm^2^ vs. CP105,696: 16.5 ± 1.3 mast cells/mm^2^; *p* = 0.80; Fig. 5B,D) and in the percentage of activated mast cells (Control: 46 ± 2% vs. CP105,696: 51 ± 2%; *p* = 0.12; Fig. 5C) in the aortic root.

## Discussion

Mast cells actively contribute to progression and destabilization of advanced atherosclerotic lesions. Prevention of mast cell recruitment to the atherosclerotic lesion could thus be a promising therapeutic strategy to limit plaque destabilization. In this study, we aimed to investigate whether inhibition of the BLT1-LTB_4_ axis via BLT1-antagonism limits mast cell accumulation in advanced atherosclerosis and via this way prevent plaque instability. Although we observed expression of genes involved in LTB_4_ biosynthesis in the human atherosclerotic plaque, including prominent expression in mast cells, inhibition of BLT1 in vivo did not affect plaque morphology and the number of mast cells in the advanced atherosclerotic plaque.

The LTB_4_ biosynthesis pathway has previously been examined in atherosclerosis. ALOX5 (5-LOX), ALOX5AP (FLAP) and LTA_4_H have been found to be expressed in human atherosclerotic plaques, and were generally seen to colocalize with macrophages^32–34^. As we show here, these genes are also detected in the murine single-cell RNA sequencing data^29^, while protein expression of ALOX5 and LTA_4_H has been established in murine plaques and adventitial macrophages as well^34^. These proteins have also been targeted in experimental atherosclerosis studies. Similar to CP105,696, promising effects on atherosclerosis development have been shown with FLAP inhibitors like MK-886 and BAYx1005^35,36^ Studies examining 5-LOX in atherosclerosis have been less evident. 5-LOX deficiency alone was not sufficient to limit atherosclerosis development, only in combination with 12–15-LO deficiency an effect was observed^37^. Furthermore, in humans, 5-LOX inhibition using VIA-2291 gave rather conflicting results. Gaztanaga et al*.* reported that treatment with VIA-2291 was not associated with significant reductions in vascular inflammation in patients after an acute coronary syndrome, while Matsumoto et al. showed that VIA-2291 resulted in slower plaque progression as measured by CT-angiography^38,39^. It must be noted that all experimental studies in mice focussed on the effects of treatment during lesion initiation. As our single-cell RNA sequencing data of advanced human plaques revealed that mast cells have high expression of the LTB_4_ biosynthesis-related genes, we aimed to examine the effect of LTB_4_ inhibition in advanced atherosclerosis.

In our study, we observed a significant reduction in splenic myeloid cells, of which the most pronounced effects were found on both the inflammatory and patrolling monocyte subsets as well as on dendritic cells. This may be a result of direct inhibition of BLT1 on these cell subsets, but may also be indirectly related as LTB_4_ has previously been shown to upregulate Monocyte Chemoattractant Protein (MCP-) 1^40^. Huang et al*.* showed that in human monocytes, LTB_4_ interacts with BLT1 to upregulate mRNA expression and active synthesis of MCP-1 by monocytes to induce a feed-forward amplification loop for their chemotaxis^41^. Furthermore, it was shown that LTB_4_ induced increased avidity and/or affinity of β1-integrin and β2-integrin to their endothelial ligands, further stimulating firm arrest of monocytes under physiologic flow^42^. Moreover, in both studies pharmacological inhibition of the LTB_4_-BLT1 axis abrogated these effects. In line with these reported findings, CP105,696 reduced splenic myeloid cell and dendritic cell levels, of which the most pronounced effects were found on inflammatory and patrolling monocytes, which both have been found to migrate towards MCP-1^43,44^.

In atherosclerosis, BLT1 has already been a target in multiple studies. In line with the chemotactic effects on monocytes, Heller et al. showed that BLT1 deficiency resulted in a significant reduction in lesion size as well as macrophage content in apoE deficient mice^15^. Furthermore, in both apoE and LDLr deficient mice, a 35-day treatment with BLT1-antagonist CP105,696 led to a significant reduction in plaque size and CD11b^+^ cells in the circulation^23^. In these studies however, intervention in the LTB1-BLT_4_ axis occurred immediately upon lesion initiation. As mast cells are known to accumulate in later stages of disease^6,9^, we aimed to assess the effect of BLT1 blockade on pre-existing plaques. However, we did not observe any differences in plaque morphology as neither plaque and necrotic core size, nor plaque collagen and macrophage content were affected by BLT1 antagonism. Furthermore, flow cytometry analysis of the atherosclerotic aortic arch did not reveal any effects on total leukocyte and myeloid cell content upon CP105,696 treatment. Apparently, blockade of BLT1 is not sufficient to affect plaque size and composition at this stage of the disease. Indeed, Aiello et al*.* described that more distinct effects of CP105,696 were observed in apoE^−/−^ mice of 15 weeks old, with smaller and thus less complex lesions as compared to 24 weeks old mice with more advanced atherosclerosis^23^. In addition, in a BLT1^−/−^apoE^−/−^ mouse model on a western type diet, differences in lesion size were only detected after 4 weeks of diet, whereas after 8 weeks and 19 weeks of diet, lesion size was similar in both BLT1^−/−^apoE^−/−^ and apoE^−/−^ mice^22^. This may be explained by the fact that monocyte influx into the plaque is a less dominant mechanism in advanced atherosclerosis as compared to early atherogenesis. In addition, macrophage egress has been shown to decrease with atherosclerosis progression^45,46^. Combined, this results in differences in myeloid cell dynamics between early and advanced atherosclerosis and effects observed upon BLT1-antagonism may thus be disease stage specific. Alternatively, in later stages of disease, other factors in the plaque microenvironment may contribute to the recruitment of different cell types to the plaque, which means that the decrease in myeloid content in the spleen due to BLT1-antagonism may not directly translate into effects in the plaque.

LTB_4_ has previously been described to induce directed migration of mast cells and their progenitors^20^. In vitro, LTB_4_ eluted from the supernatant of activated BMMCs elicited chemotaxis of immature mast cells. In mice, increased recruitment of CMFDA-labelled mast cell progenitors was detected upon injection of LTB_4_ into the dorsal skin. LTB_4_ was shown to be a potent chemoattractant for immature c-kit^+^ human umbilical cord blood-derived mast cells (CBMCs), whereas mature c-kit^+^ mast cells remained unresponsive^19^. Nevertheless, in our study we did not observe any differences in the percentage of circulating mast cell progenitors after 4 weeks of treatment with CP105,696. Moreover, the total number of mast cells and the percentage of activated mast cells in the aortic root also remained unaffected, suggesting that LTB_4_ does not act as a chemoattractant for mast cells in advanced atherosclerosis via the LTB_4_ receptor BLT1. Assessment of mast cell accumulation in other sites of advanced atherosclerosis may be warranted to confirm our findings. In vitro cultured murine and human mast cells have however also shown expression of the LTB_4_ low affinity receptor BLT2. Lundeen et al*.* showed that inhibition with a selective BLT2 antagonist dose-dependently reduced migration of mast cells towards LTB_4_ in vitro, suggesting that this interaction of BLT2 and LTB_4_ could be involved in mast cell chemotaxis^20^. As CP105,696 is a selective BLT1-antagonist, we cannot exclude BLT2-induced mast cell recruitment towards the plaque in our experiment. Future studies may aim to investigate whether BLT2 is involved in mast cell chemotaxis to the advanced plaque independent of BLT1.

Although we did not see any differences in mast cell recruitment towards the atherosclerotic plaque in CP105,696 treated mice compared to our controls, single-cell RNA sequencing of human carotid atherosclerotic lesions suggests that mast cells may contribute to the local LTB_4_ concentrations in the plaque as intraplaque mast cells highly expressed genes involved in LTB_4_ biosynthesis. Interestingly, *ALOX5*, encoding for 5-LOX, was most prominently expressed in intraplaque mast cells as compared to other plaque cell types. In line, Spanbroek et al*.* showed that 5-LOX colocalized with tryptase^+^ mast cells in carotid arteries and that the number of 5-LOX expressing cells increased in later stages of disease^47^. Furthermore, 5-LOX expression was mainly found in the shoulder regions of atherosclerotic lesions^48^, where mast cells have also been shown to reside and accumulate^49^. Together, this suggest that although mast cells may not induce an autocrine loop for their recruitment towards murine atherosclerotic lesions, that they may induce migration of other leukocytes towards the lesion via LTB_4_. We cannot exclude species-induced differences here, however based on current literature, we do not expect any differences as both mouse and human mast cells in culture were seen to migrate towards LTB_4_^19,20^.

To conclude, here we show that BLT1-antagonism does not affect plaque size and morphology during advanced stages of atherosclerosis, which suggests that LTB_4_ is not involved in the progression of advanced atherosclerotic lesions. Moreover, we show that LTB_4_ does not seem to be involved in mast cell migration towards atherosclerotic plaques, but that mast cells are able contribute to local LTB_4_ production in the lesion. To identify novel therapeutic intervention strategies, further research should be aimed at the elucidation of mechanisms that induce directed migration of mast cells towards the advanced atherosclerotic plaque.

## Methods

### Single-cell RNA sequencing

Human carotid artery plaques were collected from 18 patients (14 male, 4 female) that underwent carotid endarterectomy surgery as part of AtheroExpress, an ongoing biobank study at the University Medical Centre Utrecht (Study approval number TME/C-01.18, protocol number 03/114)^50^. Single cells were obtained and processed for single-cell RNA sequencing as previously described^28^. All studies were performed in accordance with the Declaration of Helsinki. Informed consent was obtained from all subjects involved in the study. Murine single-cell RNA sequencing data sets were obtained from Cochain et al.^29^ and processed as previously described^51,52^. Briefly, data sets were processed using the SCTransform normalization method^53^, integrated using rpca reduction and subsequently clustered, all according to the Seurat “scRNA-seq integration” vignette^54^. All data analyses were executed in R-4.0.2.

### Animals

All animal experiments were performed in compliance with the guidelines of the Dutch government and the Directive 2010/63/EU of the European Parliament. The experiment was approved by the Ethics Committee for Animal Experiments and the Animal Welfare Body of Leiden University (Project 106002017887, Study number 887, 1–103).

Female 7–10 week old LDLr^−/−^ mice (C57BL/6 background) (n = 15/group) that were bred in-house were provided with food and water ad libitum. From the start of the experiment, the mice were fed a cholesterol-rich western-type diet (0.25% cholesterol, 15% cocoa butter, Special Diet Services, Essex, UK), which continued for 9 weeks in total. At week 5, the mice were randomized in groups based on age, weight and serum cholesterol levels. Previous work from our group showed that mast cell accumulation in the aortic root starts at approximately 6 weeks after the start of western-type diet feeding^55^. Therefore, from week 5 onwards, mice received either 20 mg/kg of BLT1-antagonist CP105,696 (Sigma-Aldrich) or vehicle control (0.6% Tween 80, 0.25% methylcellulose in phosphate-buffered saline (PBS)) three times per week via oral gavage for 4 weeks (n = 15 per group). A detailed schedule of the experimental setup is provided in Figure S2. Blood was drawn by tail vein bleeding at week 5 and week 7. At week 9, the mice were sacrificed upon subcutaneous administration of anaesthetics (ketamine (40 mg/mL), atropine (0.1 mg/mL) and xylazine (8 mg/mL)). Blood was collected via orbital bleeding, after which the mice were perfused with PBS through the left cardiac ventricle. Next, organs were collected for analysis.

### Cholesterol and triglyceride assay

Serum was collected through centrifugation at 8000 rpm for 10 min at 4 °C and stored at − 80 °C until further use. Total cholesterol levels were determined through an enzymatic colorimetric assay (Roche/Hitachi, Mannheim, Germany). Triglyceride levels in serum were measured by an enzymatic colorimetric assay (Roche Diagnostics). For both assays, Precipath standardized serum (Roche Diagnostics) was used as an internal standard.

### Cell isolation

Blood samples were lysed with ACK lysis buffer (0.15 M NH_4_Cl, 1 mM KHCO_3_, 0.1 mM Na_2_EDTA, pH 7.3) to obtain a single white blood cell suspension. Spleens were passed through a 70 μm cell strainer (Greiner, Bio-one, Kremsmunster, Austria) and splenocytes were subsequently lysed with ACK lysis buffer. Aortic arches were cut into small pieces and enzymatically digested in a digestion mix containing collagenase I (450 U/mL), collagenase XI (250 U/mL), DNAse (120 U/mL), and hyaluronidase (120 U/mL; all Sigma–Aldrich) for 30 min at 37 °C while shaking. After incubation, all samples were passed through a 70 µm cell strainer (Geiner, Bio-one, Kremsmunster, Austria). Single cell suspensions were then used for flow cytometry analysis.

### Flow cytometry

Single cell suspensions from blood and spleen were extracellularly stained with a mixture of selected fluorescent labelled antibodies for 30 min at 4 °C. The antibodies used for flow cytometry are listed in Table S1. All measurements were performed on a Cytoflex S (Beckman and Coulter, USA) and analysed with FlowJo v10.7 (Treestar, San Carlos, CA, USA).

### Histology

After euthanasia, the hearts were dissected, embedded and frozen in Tissue-Tek OCT compound (Sakura). 10 μm cryosections of the aortic root were prepared for histological analysis. Mean plaque size and the percentage plaque area of total vessel area (vessel occlusion) were assessed by Oil-Red-O (ORO) staining. From the first appearance of the three aortic valves, 5 consecutive slides with 80 µm distance between the sections were analysed for total lesion size within the three valves, after which the average lesion size was calculated. Average lesion size was also calculated in relation to distance from the start of the three-valve area. Subsequently, plaque volume was calculated as area under the curve. Collagen content of the plaque was measured using a Sirius Red staining after which fluorescent staining of three section per mouse was analysed and averaged. Similarly, the average necrotic core size was measured using the Sirius Red staining by measuring the acellular debris-rich areas of the plaque of three sections per mouse. Macrophage content was determined by using MOMA-2 antibody (1:1000; rat IgG2b; Bio-Rad). Naphthol AS-D chloroacetate staining (Sigma-Aldrich) was performed to manually quantify resting and activated mast cells in the plaques. Mast cells were identified and counted in the perivascular tissue of the aortic root at the site of atherosclerosis. A mast cell was considered resting when all granula were maintained inside the cell, while mast cells were assessed as activated when granula were deposited in the tissue surrounding the mast cell. Sections were digitalised using a Panoramic 250 Flash III slide scanner (3DHISTECH, Hungary). Analysis was performed using ImageJ software.

### Cell Culture

Bone marrow-derived mast cells (BMMCs) from 7 to 10 weeks LDLr^−/−^ mice were cultured in RPMI 1640 containing 25 mM HEPES (Lonza) and supplemented with 10% fetal calf serum, 1% L-glutamine (Lonza), 100 U/mL mix of penicillin/streptomycin (PAA), 1% sodium pyruvate (Sigma-Aldrich), 1% non-essential amino acids (MEM NEAA; Gibco) and 5 ng/mL IL-3 (Immunotools). Cells were incubated at 37 °C and 5% CO_2_ and were kept at a density of 0.25*10^6^ cells per mL by weekly subculturing. BMMCs were cultured for 4 weeks in total to obtain mature mast cells.

### RNA isolation and gene expression analysis

RNA isolation from 1*10^6^ mast cells was performed using the guanine isothiocyanate method^56^. Using RevertAid M-MuLV reverse transcriptase cDNA was isolated according to the manufacturer’s instructions. Quantitative gene expression analysis was performed with the SYBR Green Master Mix technology on a QuantStudio 6 Flex (Applied Biosystems by Life Technologies). A list of qPCR primers can be find in Table S2.

### Statistical analysis

The data are presented as mean ± SEM and analysed in GraphPad Prism 9. Shapiro-Wilkson normality test was used to test data for normal distribution. Outliers were identified by a Grubbs’ test. Data was analysed using an unpaired two-tailed Student *t*-test or Mann–Whitney test. *p* < 0.05 was considered to be significant.

### Ethics declarations

All experiments have been performed in accordance with the ARRIVE guidelines.

*Human samples* The study was conducted according to the guidelines of the Declaration of Helsinki, and approved by the Medical Ethical Committee of the University Medical Centre Utrecht (UMCU). All samples were included in the Athero-Express Study (www.atheroexpress.nl), an ongoing biobank study at the UMCU. Informed consent was obtained from all subjects involved in the study.

*Animal studies*: All animal experiments were performed in compliance with the guidelines of the Dutch government and the Directive 2010/63/EU of the European Parliament. The experiment was approved by the Ethics Committee for Animal Experiments and the Animal Welfare Body of Leiden University (project 106,002,017,887, study number 887,1–103).

## Supplementary Information

Supplementary Information.

## Data Availability

The data presented in this study are available on request from the corresponding author. The human scRNAseq data presented in this study are retrieved from Depuydt et al. Circ Res. 2020^28^. R scripts are available on GitHub (https://github.com/AtheroExpress/MicroanatomyHumanPlaque_scRNAseq). Other data is available upon request from the corresponding authors of this paper.
