# Supplementary material for: Blockade of the BLT1-LTB4 axis does not affect mast cell migration towards advanced atherosclerotic lesions in LDLr−/− mice
Source: Sci Rep. 2022 Nov 1;12:18362. doi: 10.1038/s41598-022-23162-4 (PMC9626554; doi:10.1038/s41598-022-23162-4)
Supplement: Supplementary file 1 — Supplementary Information. [file 41598_2022_23162_MOESM1_ESM.docx]

Supplementary files

Blockade of the BLT1-LTB_4_ axis does not affect mast cell migration towards advanced atherosclerotic lesions in LDLr^-/-^ mice.

Marie A.C. Depuydt^1^, Femke D. Vlaswinkel^1^, Esmeralda Hemme^1^, Lucie Delfos^1^, Mireia N.A. Bernabé Kleijn^1^, Peter J. van Santbrink^1^, Amanda C. Foks^1^, Bram Slütter^1^, Johan Kuiper^1^ and Ilze Bot^1,^*

^1^Division of BioTherapeutics, Leiden Academic Centre for Drug Research, Leiden University, Leiden, The Netherlands.

*Correspondence: [i.bot@lacdr.leidenuniv.nl](mailto:i.bot@lacdr.leidenuniv.nl)


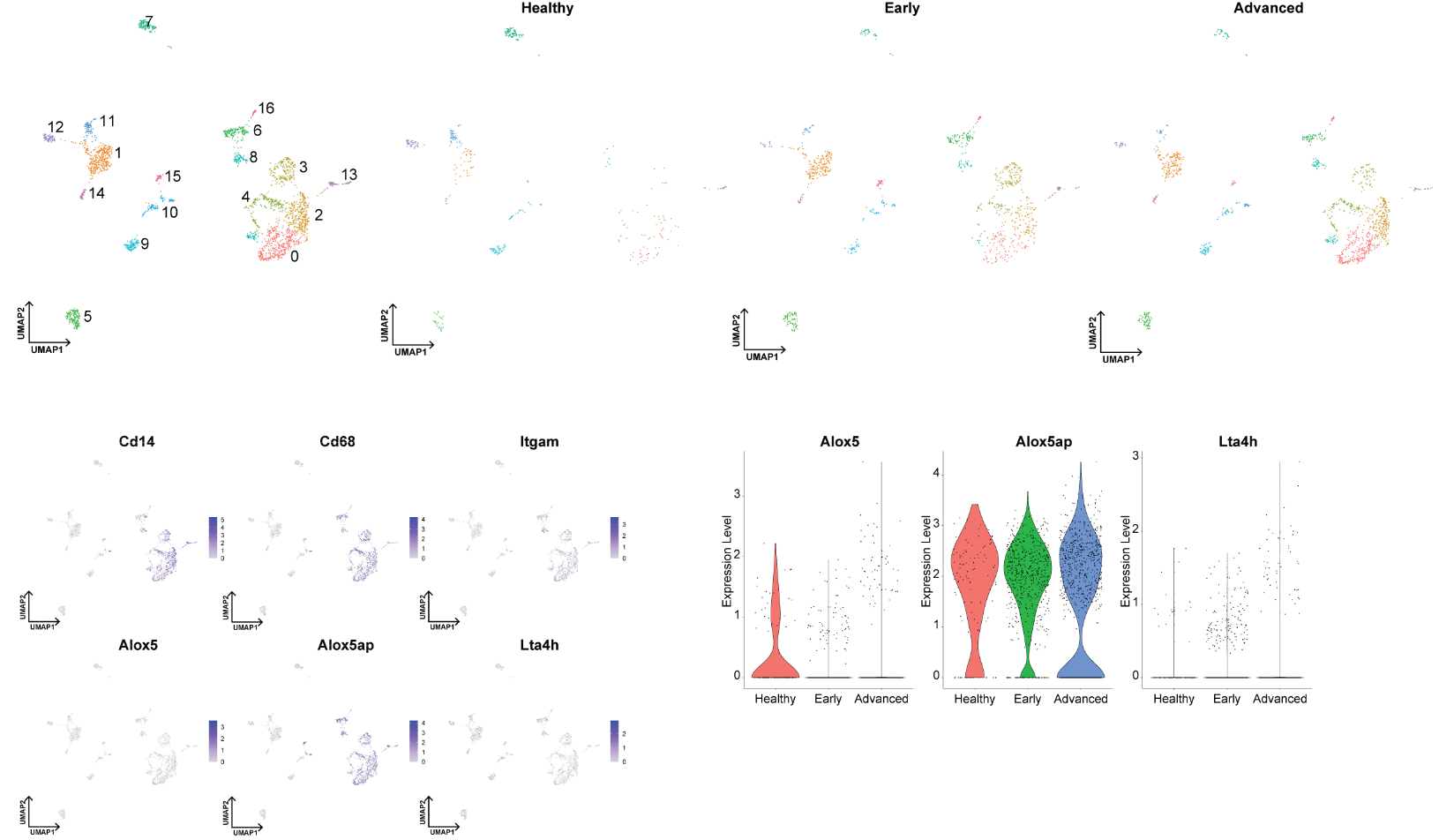


**B**

**A**

**Figure S1**. Single-cell RNA sequencing reveals expression of Alox5, Alox5ap, and Lta4h in healthy (chow diet), early atherosclerotic (11wk HFD) and advanced atherosclerotic aortas (20wk HFD) of LDLr^-/-^ mice.^29^ (A) Integrated analysis of CD45+ cells of aorta’s from LDLr^-/-^ in different plaque stages reveals UMAP with 17 clusters. (B) UMAP depicting distribution of CD45^+^ cells of healthy, early atherosclerotic and advanced atherosclerotic aortas. (C) Alox5, Alox5ap and Lta4h are mainly expressed in myeloid cell clusters (Cd14^+^, Cd68^+^, Itgam^+^). (D) Expression of Alox5, Alox5ap and Lta4h in all myeloid cell clusters divided by plaque stage.

**D**

**C**


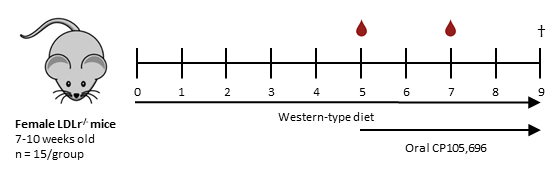


**Figure S2**. Schematic overview of the experimental set-up. Female *LDLr^-/-^* were put on a western type diet for 9 weeks and were treated with 20mg/kg CP105,696 or vehicle control three times a week by oral gavage from week 5 up to week 9. Blood was drawn via tail vein bleeding at week 5 and week 7. At week 9, all mice were sacrificed, after which organs were collected and processed for analysis.

**Figure S3**. The degree of stenosis and plaque volume did not differ upon treatment with CP105,696. Oil red O staining did not reveal differences in (A) the degree of stenosis (percentage plaque area of total vessel area), (B) the lesion area and (C) plaque volume between the CP105,696 versus vehicle control mice. Data represents mean ± SEM.

**A**

**B**

**C**

**Figure S4.** BLT1-antagonism did not affect the percentage of myeloid cells in the aortic arch. A) Flow cytometry analysis of the aortic arch revealed no differences in the percentage of aortic leukocytes (live CD45^+^) within the single cell population upon treatment with CP105,696. Furthermore, the percentages of B) CD11b^+^ myeloid cells and C) CD11b^+^CD11c^+^ dendritic cells within the CD45^+^ cell population did not differ between groups. n = 14-15 per group. Data represent mean ± SEM.

**A**

**B**

**C**

**Table S1.** Extracellular antibodies used for flow cytometry analysis.

| **Antibody** | **Fluorochrome** | **Company** | **Cat. No.** | **Clone** | **Dilution** |
| --- | --- | --- | --- | --- | --- |
| CD16/32 | Brilliant violet 421 | Biolegend | 101331 | 93 | 1:400 |
| CD127 | Brilliant violet 605 | Biolegend | 135025 | A7R34 | 1:400 |
| CD11c | FITC | Biolegend | 117306 | N418 | 1:800 |
| CD45R | FITC (Lineage) | Biolegend | 103206 | RA3-6B2 | 1:400 |
| CD11b | FITC (Lineage) | eBioscience | 11-0112-85 | M1/70 | 1:400 |
| Gr-1 | FITC (Lineage) | eBioscience | 11-5931-82 | RB6-8C5 | 1:400 |
| CD3 | FITC (Lineage) | eBioscience | 11-0031-85 | 145-2C11 | 1:400 |
| Ter119 | FITC (Lineage) | Biolegend | 116206 | TER-119 | 1:400 |
| CD19 | FITC (Lineage) | eBioscience | 11-0191-85 | MB19-1 | 1:400 |
| CD4 | FITC (Lineage) | eBioscience | 11-0041-85 | GK1.5 | 1:400 |
| Sca-1 | PE | BD Biosciences | 553336 | E13-161.7 | 1:400 |
| CD11b | PE | Biolegend | 101208 | M1/70 | 1:1000 |
| Ly6C | PE-CF594 | BD Biosciences | 562728 | AL-21 | 1:800 |
| CD34 | PE-Cy7 | Biolegend | 119325 | MEC14.7 | 1:400 |
| cKit/CD117 | APC | eBioscience | 17-1171-82 | 3B8 | 1:400 |
| Fcer1 | AF700 | Biolegend | 134324 | MAR-1 | 1:400 |
| Fixable Viability dye | eFluor780 | ThermoFisher Scientific | 65-0865-14 | n/a | 1:2000 |
| Fc Block | n/a | Biolegend | 101320 | n/a | 1:250 |

**Table S2.** Primers used for quantitative reverse transcription PCR (RT-qPCR). For all analyses, *36b4* and *Rpl27* were included as housekeeping genes.

| **Gene** | **Forward primer** | **Reverse Primer** | **Size** | **Accession number** |
| --- | --- | --- | --- | --- |
| *36b4* | ctgagtacaccttcccacttactga | cgactcttcctttgcttcagcttt | 885-1033 | NM_007475.5 |
| *Rpl27* | cgccaagcgatccaagatcaagtcc | agctgggtccctgaacacatccttg | 400-529 | NM_011289.3 |
| *Kit* | tggtcaaaggaaatgcacgactgcc | catccctgggtaggggctgcttc | 2542-2690 | NM_001122733.1 |
| *Alox5* | gcagatcgtggatactctaccagacc | aaatgctcctctgggtacatgcctag | 1912-2031 | NM_009662.2 |
| *Alox5ap* | aagcaagcatggatcaagaggctgtg | aagcttctcccattatgcgccttgc | 102-234 | NM_009663.2 |
| *Lta4h* | acggctctgcattcaatcgaaatgg | ggacagcttgatcatgggatttgtcg | 1734-1873 | NM_008517.2 |
